# PReS-FINAL-2181: Recombinant il-1ra restores the il-18-nk cell axis in steroid naive systemic juvenile idiopathic arthritis

**DOI:** 10.1186/1546-0096-11-S2-O16

**Published:** 2013-12-05

**Authors:** W De Jager, SJ Vastert, G Mijnheer, BJ Prakken, NM Wulffraat

**Affiliations:** 1 Pediatric Immunology, university medical center utrecht, Utrecht, Netherlands

## Introduction

Systemic onset juvenile idiopathic arthritis (sJIA) is an acquired auto-inflammatory disease characterized by systemic inflammation and innate immune activation reflected in uncontrolled production of cytokines such as IL-1, IL-6 and IL-18. In sJIA, NK cell function is severely hampered despite high levels of IL-18. We recently found that defective phosphorylation of the IL-18 receptor beta is responsible for the deficient IL-18-NK cell axis in sJIA.

## Objectives

Given the strong homology between the IL-1 and the IL-18 receptor we questioned whether treatment with rIL-1RA (Anakinra) may directly down regulate IL-18 production and restore the IL-18 signaling cascade in NK cells and, if so, whether this relates to the clinical improvement observed in sJIA patients. Therefore we treated 15 consecutive patients with newly onset sJIA, before start of steroids.

## Methods

Clinical (ACRped) and laboratory parameters (NK Cell activity, cytokine levels) were analyzed during 90 days after initial start of rIL-1RA treatment. To study binding interaction between rIL-1RA and both the IL-1R and the IL-18R a human cell line (KG1) was used.

## Results

We show that patients with sJIA have increased inflammasome activation leading to elevated IL-18 levels. Treatment with recombinant rIL-1RA in steroid naïve newly onset sJIA patients led to rapid resolution of clinical features in 87% of patients. *In vitro*, rIL-1RA directly antagonized IL-18 signaling and lead to normalization of both inflammasome activation and IL-18 levels. Finally, *in vivo *first line treatment with rIL-1RA in sJIA patients led to a normalization of both IL-18 levels and inflammasome activation and a restoration of the deficient IL-18-NK cell axis, correlating with a favorable clinical response in these patients.

## Conclusion

The rapid clinical efficacy of early treatment with rIL-1RA in sJIA is accompanied by a restoration of the IL-18-NK-cell axis in sJIA. These data provide biological support for the use of rIL-1RA sJIA patients early in the disease course as first line treatment, as it directly interferes with the deficient IL-18-NK cell axis which may lead to a intrinsically change in the course of the disease.

## Disclosure of interest

None declared.

